# Predicting disease-related genes using integrated biomedical networks

**DOI:** 10.1186/s12864-016-3263-4

**Published:** 2017-01-25

**Authors:** Jiajie Peng, Kun Bai, Xuequn Shang, Guohua Wang, Hansheng Xue, Shuilin Jin, Liang Cheng, Yadong Wang, Jin Chen

**Affiliations:** 1 School of Computer Science, Northwestern Polytechnical University, Xi’an, China; 2 School of Computer Science and Technology, Harbin Institute of Technology, Harbin, China; 3 Department of Mathematics, Harbin Institute of Technology, Harbin, China; 4 College of Bioinformatics Science and Technology, Harbin Medical University, Harbin, China; 5 Institue of Biomedical Informatics, College of Medicine, University of Kentucky, Lexington, 40536 KY USA; 6 Department of Energy Plant Research Lab, Michigan State University, East Lansing, 48824 MI USA; 7 Current address: Tencent, Inc., Shenzhen, China

**Keywords:** Disease gene prediction, Laplacian normalization, Supervised random walk, Integrated network

## Abstract

**Background:**

Identifying the genes associated to human diseases is crucial for disease diagnosis and drug design. Computational approaches, esp. the network-based approaches, have been recently developed to identify disease-related genes effectively from the existing biomedical networks. Meanwhile, the advance in biotechnology enables researchers to produce multi-omics data, enriching our understanding on human diseases, and revealing the complex relationships between genes and diseases. However, none of the existing computational approaches is able to integrate the huge amount of omics data into a weighted integrated network and utilize it to enhance disease related gene discovery.

**Results:**

We propose a new network-based disease gene prediction method called SLN-SRW (Simplified Laplacian Normalization-Supervised Random Walk) to generate and model the edge weights of a new biomedical network that integrates biomedical data from heterogeneous sources, thus far enhancing the disease related gene discovery.

**Conclusions:**

The experiment results show that SLN-SRW significantly improves the performance of disease gene prediction on both the real and the synthetic data sets.

**Electronic supplementary material:**

The online version of this article (doi:10.1186/s12864-016-3263-4) contains supplementary material, which is available to authorized users.

## Background

One crucial step toward understanding the molecular basis of diseases, such as cancer, diabetes, and cardiovascular disorders, is to identify the predisposing or virulence genes of these diseases, which will lead to early disease diagnosis and effective drug design [1]. With the availability of the big biomedical data, researchers tend to get insights into human diseases by identifying genes that might cause or relate to them. Given the fact that experimentally identifying of the complete list of disease-related genes is generally impractical due to the high cost, computational methods have been proposed in the last decades to predict the relationships between genes and human diseases [2–10]. However, these tools, including filtering methods based on a set of criteria [11], text mining of biomedical literature [12], integration of genomic data [13–15], semantic similarity [16–21] based disease gene prioritization [22] and network analysis based and highly robust approach [8, 23–26], remain pre-eminent [27].

A human cell consists of several functionally inter-dependent molecular components. A human disease rarely results from an abnormality in a single gene but reflects the perturbations of the complex molecular network induced by different kinds of factors, such as genetic variations, pathogens and epigenetic changes [28]. The molecular network links molecular states to physiological states associated with diseases in a whole system view [29]. Therefore, network-based approaches may offer better targets for drug development, and may lead to multiple potential biological and clinical applications including disease gene discovery [28].

The network-based approaches for disease gene identification can be loosely grouped into three categories. The simplest approach, named direct neighbor counting, is to check whether two genes are connected directly in a molecular network. The idea is that if a gene is connected to one of the known disease genes, it may be associated with the same disease [30]. The experimental result demonstrates that using molecular networks can effectively increase the likelihood of identifying candidate disease genes. The direct neighbor counting method, however, does not consider the situation that two genes are not connected directly but still have certain biological associations. To address this problem, Kruthammmer et al. employed the shortest path length approach to measure the closeness between a disease gene and a candidate gene. This method has been successfully applied to predict the genes associated Alzheimer’s disease, and the prediction results agree with the manually curated candidates [31]. Since both the direct neighbor counting method and the shortest path method are local distance measurements, they largely ignore the global structure of the whole molecular network and cannot fully capture the complex relationships between network nodes [32]. Subsequently, methods have been proposed to predict the gene-disease relation using the global network structure, such as random walk with restart (RWR) [33], propagation flow [34], Markov clustering [35] and graph partitioning [36]. The performance evaluation on HPRD [37], OPHID [38] and OMIM [39] dataset shows that RWR is the best among the then-existing methods [5].

Rapidly evolving bio-technologies promote collecting multiple types of biological data, including diverse genome-scale data, clinical phenotype data, environment data, and data of daily activities [40], making it feasible and attractive to build integrated biomedical networks from multiple sources, rather than focus on one single data set. The integrated network that includes multiple, heterogeneous types of resources, greatly extends the scope and ability for disease gene prediction [41]. For example, BioGraph [42] uses data from 21 publicly available curated databases to identify relations between heterogeneous biomedical entities. The work by Ganegoda et al. runs RWR on a integrated network, and has successfully identified disease-related genes with significant improved performance [43].

Using integrated networks for gene-disease relationship discovery is still a difficult task due to the existence of multiple biomedical entities in the integrated networks. In a network built using a single type of biomedical data, there is only one type of nodes and one type of edges. For example, in a protein-protein interaction network, nodes and edges represent proteins and protein interactions respectively. The integrated network, on the contrary, contains multiple types of nodes and edges representing different biomedical entities (such as genes, diseases, and ontology terms) and relationships (such as DNA-protein binding and gene ontology annotation). In order to differentiate these edge types, edge weights in the integrated biomedical network should be appropriately assigned [44].

In this article, we present a new algorithm called SLN-SRW (Simplified Laplacian Normalization-Supervised Random Walk) to define edge weights in an integrated network and use the weighted network to predict gene-disease relationships. Comparing with the existing approaches, SLN-SRW has the following advantages:

- SLN-SRW is the first approach, to the best of our knowledge, to predict gene-disease relationships based on a weighted integrated network with its edge weight being computed to precisely describe the importance of different edge types.
- The performance of random walk may be strongly affected by the super hub nodes in an integrated network. SLN-SRW adopts a Laplacian normalization based method to avoid such bias.
- To prepare inputs for SLN-SRW, we constructed a new heterogeneous integrated network based on three widely used biomedical ontologies, i.e. Human Phenotype Ontology [45], Disease Ontology [46], and Gene Ontology [47, 48], and biological databases such as STRING [49]. This integrated network combines biomedical knowledge from ontologies that are manually curated and big biomedical data that have been deposited in databases. Based on these two distinctively different types of information, this network forms a foundation for disease gene discovery.

## Methods

**Fig. 1:**
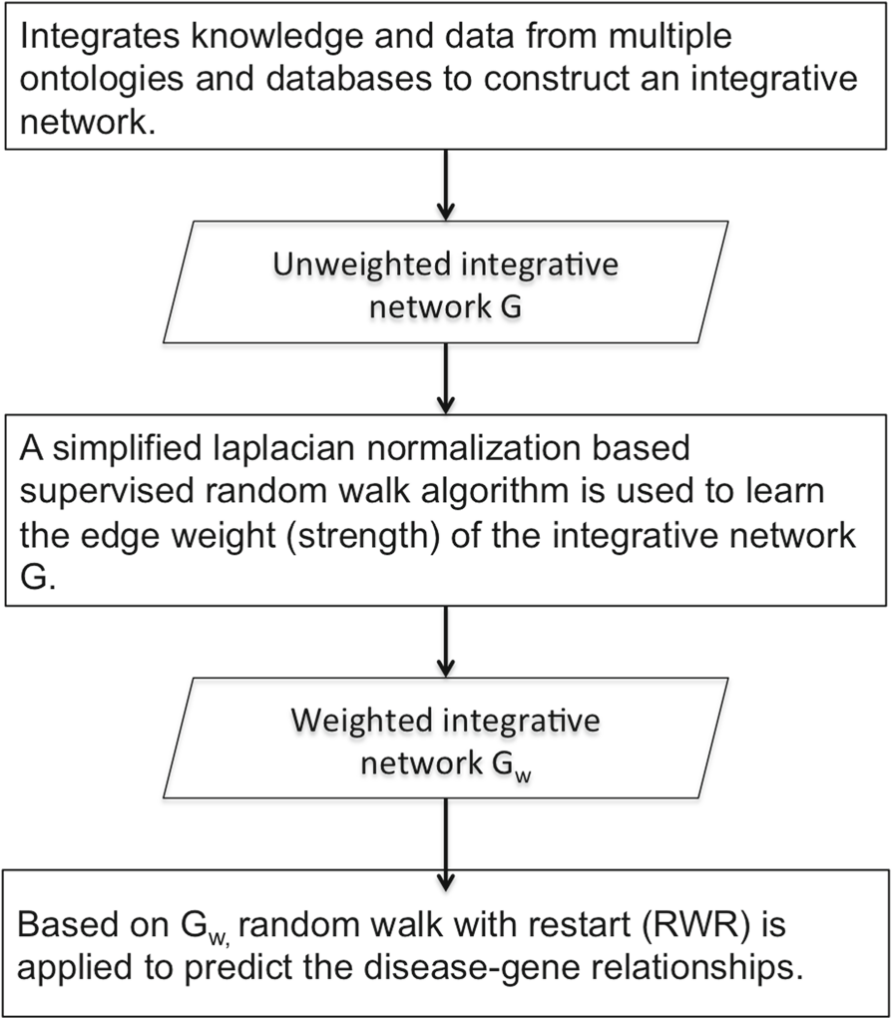
The Framework of *SLN*−*SRW*. Framework of SLN-SRW for estimating the edge weight of the integrated network automatically and predicting disease genes based on it. The second step is the essential part of SLN-SRW algorithm

We propose SLN-SRW to compute and model the edge weight of an integrated network and then predict disease genes. To achieve the goal, SLN-SRW consists of three steps. First, it integrates knowledge and data from multiple ontologies and databases to construct an integrated network *G*(*V*,*E*), where *V* is a set of nodes and *E* is a set of edges that connect the nodes in *V*. Second, it uses a Laplacian normalization based supervised random walk algorithm to learn the edge weight of network *G*, resulting in a weighted integrated network *G*_*w*_. Third, it employs the RWR method on *G*_*w*_ to predict disease-gene relationships. The diagram of the whole process of SLN-SRW is shown in Fig. 1. We will introduce the key steps of SLN-SRW in the rest of this section.

### Step 1. Integrating heterogeneous knowledge and data sources for integrated network construction

In the first step of SLN-SRW, an integrated network is constructed based on eleven heterogeneous data sources in four distinct forms, i.e. ontologies, networks, unified vocabularies, and relational databases. The data sources are listed in Table 1, and they are mainly used for relation extraction, name mapping, and unified vocabulary. The data sources can be grouped into two categories: 1) Curated data that were collected from literature and other high quality data sources, such as Search Tool for the Retrieval of Interacting Gene/Proteins (STRING) and Online Mendelian Inheritance in Man (OMIM), and 2) Curated ontologies that constructed manually by the domain expert, such as Gene Ontology (GO) and Disease Ontology (DO).

**Table 1:** Integrated databases and ontologies. The first column, second column, and third column represent the abbreviation of the data source, simplify the description of the data source and the relationship extracted from the data source respectively. Eleven data sources are used to construct the integrated network. Specific types of nodes and edges are extracted from various data sources and integrated into a network

Abbreviation	Data sources	Relationship
STRING	Search Tool for the Retrieval of Interacting Gene/Proteins	gene-gene
CTD-DG	The Comparative Toxicogenomics Database - Curated Disease-Gene Interactions	disease-gene
OMIM	Online Mendelian Inheritance in Man Disease Subtypes	disease-gene
ClinVar	Clinical Variants and phenotypes	Disease/Phenotype-gene
HGNC	HUGO gene Nomenclature Committee Database	gene name mapping
MeSH	Medical Subject Headings	Unified vocabulary
UMLS	Unified Medical Language System	Unified vocabulary
SIDD	Semantically Integrated Disease-associated Database	disease name mapping
DO	Human Disease Ontology	DO term-gene/ DO term-DO term
HPO	Human Phenotype Ontology	HPO term-gene/ HPO term-HPO term
GO	Gene Ontology	GO term-gene/GO term-GO term

**Fig. 2:**
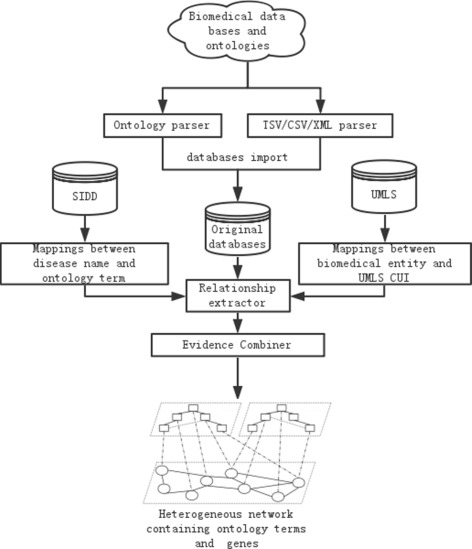
The workflow of constructing the integrated network. Work flow of constructing the integrated network based on multiple data sources
- **Unifying biomedical entity IDs.** The same pair-wise relation may be extracted from multiple data sources with different identifiers. To avoid confusion, we provide a distinct ID number for each biomedical entity by mapping all types of identifiers to the ones provided in the Unified Medical Language System (UMLS). The challenge is that some types of identifiers cannot be direct mapped to UMLS. For example, only a small part (61%) of HPO and DO term can be mapped to UMLS. Therefore, we adopted ClinVar [50] to map all the HPO terms to UMLS, and utilized SIDD [51] to map all the disease names in DO to MeSH ID, provided that there are direct mappings between MeSH ID and UMLS. Please see Additional file 1 for more details. After unifying the entity IDs from multiple data sources, each entity only has one identifier in the database. We removed the identifiers that cannot be mapped to UMLS.
- **Constructing the integrated network.** The binary relations extracted from multiple data sources form an integrated network *G*, in which nodes are biomedical entities (i.e. ontology terms and genes), and edges are the relationships between the entities, which have seven different types: GO term - GO term, GO term - gene, DO term - DO term, DO term - gene, HPO term - HPO term, HPO term - gene, and gene - gene.
- **Edge initial weight assignment.** We assign the initial edge weight *t*(*u*,*v*) to every edge <*u*,*v*> according to its edge type and the evidence code associated to the edge, where both *u* and *v* are nodes in *G*. Specifically, for the edge types that have edge confidence scores in the source databases, we use the confidence scores directly. For the edge types that do not have confidence scores but are associated with evidence codes, we manually assign initial edge weights based on their edge evidence codes (see Additional file 2 for the manually assigned weights). The edge initial weights are between 0 and 1, and the experimentally verified edges have higher initial weights than computational predictions. For example, an edge between a GO term and a gene with evidence code “EXP” has a high weight (1.0), whereas an edge with “IEA” code has a low weight (0.4), since “EXP” indicates the GO-gene relationship has been experimentally verified while “IEA” means computational prediction. Note that for the edges that have two or more evidences in *E*, the initial weights are calculated as the maximal weight of all the valid evidence codes.

The workflow for constructing the integrated network out of the heterogeneous data sources is shown in Fig. 2. Specifically, the network construction process has the following four steps:

- **Extracting information from heterogeneous data sources.** Ontology parser and database parser have been developed for ontology and database data extraction respectively. The ontology parser processes the OBO file and the ontology annotation file, since HPO, DO and GO are all in Open Biomedical Ontologies (OBO) format. The database parser processes files in Tab Separated Values (TSV), Comma Separated Values (CSV), and Extensible Markup Language (XML) format. The outputs of the two parsers are pair-wise relations and their properties between two biomedical entities.

### Step 2. Weighing the importance of different types of edges in integrated network

Given an integrated network *G* with manually assigned initial edge weights, the aim of this step is to automatically re-assign all the edge weights, such that the weighted network *G*_*w*_ can be used for more precise disease gene prediction. To achieve this goal, we develop a new edge weight optimization algorithm based on supervised random walks (SRW) [52]. SRW combines the information from network structure with the node and edge level attributes, which can guide the random walk on the network. By running SRW, we expect to re-assign weights to all the edges, such that the random walker from a disease node is more likely to visit the nodes representing the associated genes. However, the training process of supervised random walks (i.e. RWR) can be significantly affected by the hub nodes in the network. To solve this problem, we propose a Laplacian normalization method to weigh the importance of different types of edges in an integrated network described as follows.

Given an integrated network *G*(*V*,*E*), let node *v*_*d*_∈*V* represent a kind of disease and let *V*_*g*_⊂*V* be the set of the candidate genes of *v*_*d*_, then the disease gene prediction problem can be converted to a problem to predict all the new edges between *v*_*d*_ and a subset of nodes in *V*_*g*_, where a critical step is to weigh the edges such that a random walker from *v*_*d*_ has higher probabilities to reach the known disease genes in *V*_*g*_ than to reach the other genes. To provide the training set for learning the edge weight, we generate a positive set *V*_*p*_ and a negative set *V*_*n*_ for every disease node *v*_*d*_, where *V*_*p*_ includes known disease genes associated with *v*_*d*_ and *V*_*n*_ includes genes not associated with *v*_*d*_.

The approach to weigh the importance of different edge types consists of the following three steps:

- **Laplacian normalization on edge weights.** To avoid the biases caused by the hub nodes in the integrated network, we adopt the Laplacian normalization method [53] to normalize all the edge weights. Given a edge (*u*,*v*)∈*E*, the edge weight of edge (*u*,*v*) is normalized by all the edges connecting to node *u* or node *v*. Mathematically, the laplacian normalized edge weight *a*(*u*,*v*) is defined as:
  1 $$ a(u,v) = \frac{f(u,v)}{\sqrt{\sum_{i \in N(u)}f(u,i)\sum_{j \in N(v)} f(v,j)}}   $$
  where *N*(*x*) is the set of neighbors of node *x*; *f*(*x*,*y*)=1/(1+*e* ^−*w*·*t*(*x*,*y*)^); *w* is the edge type importance vector for graph *G* that we will learn in the next step using an optimization process, and its length is equal to the number of possible edge types (in our case, seven); *t*(*x*,*y*) is the vector of the initial weight of edge <*x*,*y*>, which has the same length as *w*. *t*(*x*,*y*) is all zero except one cell because each edge can have one and only one kind of edge type. Note that the edge type is decided by the type of nodes connected by it. For example, gene - gene and HPO term - gene are two different types of edges in the integrated network. *a*(*u*,*v*) integrates and normalizes both the edge type importance *w* and the initial edge weight *t*; it can be used to model the random walk transition probability.
- **Edge weight optimization - problem formation.** In order to learn the optimal *w* for all the seven edge types in an integrated network, we minimize an optimal function defined in Eq. 2, such that the random walker in the network is more likely to reach the genes in *V*_*p*_ than the genes in *V*_*n*_.
  2 $$ {{} {\begin{aligned} w&=\arg\min_{w} O(w) \\&\quad\,=\,\ \arg\min_{w}\left(\frac{1}{2}{||w||}^{2} \,+\, \lambda\sum\limits_{v_{d} \in D}\sum\limits_{v_{p}\in V_{p}, v_{n} \in V_{n}} h\left(S_{v_{n}}\,-\,S_{v_{p}}\right)\right)  \end{aligned}}}  $$
  where ||*w*|| is the euclidean norm; and *D* is a set of starting nodes representing the diseases in the training set. For each disease node *v*_*d*_∈*D*, *V*_*p*_ and *V*_*n*_ representing the positive training set and the negative training set respectively. $S_{v_{p}}$ ($S_{v_{n}}$) is the association value between *v*_*d*_ and *v*_*p*_∈*V*_*p*_ (*v*_*d*_ and *v*_*n*_∈*V*_*n*_), which can be calculated by running RWR on *G* [54]. *λ* is the weight penalty score deciding to what extent the constraints can be violated. Given the value of $S_{v_{n}}-S_{v_{p}}$, *h*() is a loss function that returns a non-negative value:
  3 $$ h(x)= \left\{ \begin{aligned} 0, ~~~~~~ x < 0 \\ \frac{1}{1+ e^{-\frac{x}{b}}}, ~~~~~~x \geq 0\\ \end{aligned} \right.  $$
  where *b* is a constant positive parameter, $x=S_{v_{n}}-S_{v_{p}}$. The smaller the *b* is, the more sensitive the loss function is (see Additional file 3). If $S_{v_{n}}-S_{v_{p}}<0$, the association between a disease and a gene in the positive training set is stronger than the association between the same disease and a gene in the negative training set, so *h*()=0. Otherwise, the constraint is violated, so *h*()>0.
- **Edge weight optimization - our solution.** To optimize edge type importance parameter *w* to minimize Eq. 2, we adopt a widely used meta-heuristics method called the gradient based optimization method [20], which has been successfully adopted to solve the link prediction problem in social networks and collaboration networks [52].
  To make the story complete, we briefly describe the gradient-based optimization method in the following text.
  First, we construct a stochastic transition matrix *Quv*′ of RWR using Eq. 1.
  4 $$ Q_{uv}'= \left\{ \begin{aligned} \frac{a(u,v)}{\sum_{w}a(u,v)}, ~~~~~~if(u,v) \in E \\ 0, ~~~~~~otherwise\\ \end{aligned} \right.  $$
  And then, based on the transition matrix *Quv*′, RWR can be described as:
  5 $$ Q_{uv} = (1-\alpha)Q_{uv}' + \alpha\mathbf{1}(v=s)  $$
  where *u* and *v* represent two arbitrary nodes in *G*; *α* is the restart probability, which is a user given threshold (in this case, we find the best value based on the training data set); and node *s* is a disease node, which is the starting node of random walk. Let $p_{i}^{(k)}$ be the probability to reach node *i* from *s* after *k* iterations. The probability vector at the *k*th iteration can be represented as $P^{(k)} = (p_{1}^{(k)}, p_{2}^{(k)},..., p_{|V|}^{(k)})^{T}$. The stationary probability vector *P*, which can be obtained after certain iterations, is the solution of the following equation:
  6 $$ P^{T} = P^{T}Q   $$
  The next step is to apply a gradient based method to identify *w* to minimize *O*(*w*) in Eq. 2. The derivative of *O*(*w*) can be calculated as follows.
  7 $$ \begin{aligned} \frac{\partial O(w)}{\partial w} &= 2w + \sum\limits_{v_{n},v_{p}}\frac{\partial h(S_{v_{n}}-S_{v_{p}})}{\partial w} \\&= 2w + \sum\limits_{v_{n},v_{p}}\frac{\partial h(S_{v_{n}}-S_{v_{p}})}{\partial (S_{v_{n}}-S_{v_{p}})}\left(\frac{\partial S_{v_{n}}}{\partial w} - \frac{\partial S_{v_{p}}}{\partial w}\right)  \end{aligned}  $$
$\frac {\partial S_{v_{x}}}{\partial w}$ can be calculated as follows:
  8 $$ \frac{\partial S_{v_{x}}}{\partial w} = \sum\limits_{v_{i}}Q_{v_{i}v_{x}}\frac{\partial S_{v_{i}}}{\partial w}+S_{v_{i}}\frac{\partial Q_{v_{i}v_{x}}}{\partial w}  $$
  This derivative can be repeatedly computed based on the estimate obtained in the previous iteration. The iteration stops when $\frac {\partial S_{v_{i}}}{\partial w}$ and $S_{v_{i}}$ do not change. The initial value of $\frac {\partial S_{v_{i}}}{\partial w}$ is 0. The $S_{v_{i}}$ is initialized as $\frac {1}{|V|}$. The initialization process is the same as the traditional SRW method. $\frac {\partial Q_{v_{i}v_{x}}}{\partial w}$ can be calculated as follows.
  Particularly, $\frac {\partial Q_{v_{i}v_{x}}}{\partial w} = 0$, if edge (*v*_*i*_,*v*_*x*_) does not exist in the network.
  9 $$ \begin{aligned} \frac{\partial Q_{v_{i}v_{x}}}{\partial w} = (1-\alpha)\frac{\frac{\partial a(v_{i},v_{x})}{\partial w}\left(\sum_{v_{j}}a(v_{i},v_{j})\right) - a(v_{i},v_{x})\sum_{v_{j}}\frac{\partial a\left(v_{i},v_{j}\right)}{\partial w}} {\left(\sum_{k}a(v_{i},v_{j})\right)^{2}} \end{aligned}  $$
  10 $$ \frac{\partial a(v_{i},v_{x})}{\partial w} = \frac{\frac{\partial f(v_{i},v_{x})}{\partial w}\pi(f(v_{i},v_{x})) - f(v_{i},v_{x})\frac{\partial \pi(f(v_{i},v_{x}))}{\partial w}} {\pi(f(v_{i},v_{x}))^{2}}  $$
  where *π*(*f*(*v*_*i*_,*v*_*x*_)) and $\frac {\partial \pi (f(v_{i},v_{x}))}{\partial w}$ are:
  11 $$ \pi(f(v_{i},v_{x})) = \sqrt{\sum_{v_{j} \in N(v_{i})}f(v_{i},v_{j})\sum_{v_{y} \in N(v_{x})}f(v_{x},v_{y})}  $$
  12 $$ \begin{aligned} \frac{\partial \pi(f(v_{i},v_{x}))}{\partial w} \,=\, \frac{\sum_{v_{j}\in N(v_{i})}\sum_{v_{y} \in N(v_{x})}\left(\frac{\partial f(v_{j},v_{i})}{\partial w}f\left(v_{y},v_{x}\right) \,+\, \frac{\partial f(v_{y},v_{x})}{\partial w}f(v_{j},v_{i})\right)} {2\sqrt{\sum_{v_{j} \in N(v_{i})}f(v_{j},v_{i})\sum_{v_{y}\in N(v_{x})}f(v_{y},v_{x})}} \end{aligned}  $$
  where *N*(*v*) is the set of neighbors of node *v*. After we get the solution of Eq. 7, we can apply a gradient descent based method and minimize *O*(*w*).

**Fig. 3:**
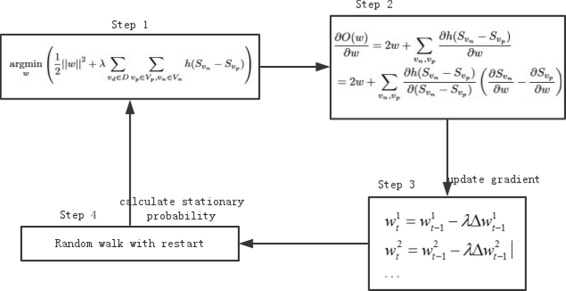
The process of training the the parameter *w*. The steps of training the the parameter *w*

Practically, the process of obtaining *w* has four steps (Fig. 3). Firstly, we initial the *O*(*w*) based on the initial parameters. Secondly, the derivative $\frac {\partial O(w)}{\partial w}$ is calculated in step 2. The power iteration is applied to calculate $\frac {\partial S_{v_{i}}}{\partial w}$ and $\frac {\partial Q_{v_{i}v_{x}}}{\partial w}$ respectively. Thirdly, based on the derivative, we can update the gradient to obtain an updated parameter *w*. Fourthly, taking the updated *w* as input, step 4 calculates the stationary probability of the RWR. In the process, the iteration for derivative calculation (step 2 in Fig. 3) and the RWR algorithm (step 4 in Fig. 3) are the two key steps. After estimating the edge weight of the integrated network, we can directly apply RWR on the weighted integrated network to predict the relation between diseases and genes.

## Results and discussion

We compare SLN-SRW with SRW and RWR, where the latter has been widely used in network-based disease gene prediction, on a real and a synthetic data sets. SLN-SRW was implemented with Java 7 in Linux.

### Data preparation

As shown in Table 1, eleven data sources, i.e. STRING [49], CTD [55], OMIM [56], ClinVar [50], HGNC [57], MeSH [58], UMLS [59], SIDD [51], DO [60], HPO [61] and GO [62], are used to construct the integrated network *G*, which has 78,786 nodes and 504,517 edges.

To test the performance of SLN-SRW, we select 430 disease-gene edges from the integrated network as the positive set. The rules for data selection are similar to the rules used in [42]. In the positive set, there are 16 diseases, each of which has at least five known disease-associated genes in the integrated network. More detail about the positive set is listed in Additional file 4. The disease-gene pairs included in the negative set are generated in two steps. First, we select a disease *d* from the positive set. Second, we repeatedly and randomly select genes that do not connect to *d* in the integrated network *G*. The number of the randomly selected genes is the same as the number of genes that connect to *d* in the positive set. We repeat the process until all disease nodes in the positive set are elected. Note that the positive set is removed from the integrated network in the testing process. Both positive and negative sets are evenly divided into two parts randomly, one for training and the other for testing.

A synthetic data set is generated following the rules in [52]. Specifically, we generated a scale-free network with 1,000 nodes using the Copying model [63] The generation process starts with three connected nodes. We connect a new node *u* to any of the existing nodes, which are selected at random with probability 0.8 or with probability proportional to the node degree. Parameter *b* is equal to 0.03 in all the experiments. For each edge in the network, we set *w*={1,−1} as the gold standard labeled as *w* ^′^. Then, we randomly choose one of the original three nodes as the start point *v*. Based on the edge strength determined by *w* ^′^, we run RWR starting from *v* and ranked the other nodes via the stationary probability. We select the top 20 nodes that directly connect with *v* as the positive training set, and select the nodes that do not connect with *v* are the negative set. Note that both the positive set and the negative set are removed from the integrated network in the testing process. In the subsection “Performance evaluation on synthetic data set”, we test whether *w* ^′^ can be estimated precisely.

### Disease gene prediction

The parameters in SLN-SRW and SRW method are estimated based on the training set. The RWR method does not need the training set for edge weight assignment. Alternatively, the training set is used to estimate the best restart probability in RWR. Finally, the performance of all the three methods is tested based on the testing set.

**Fig. 4:**
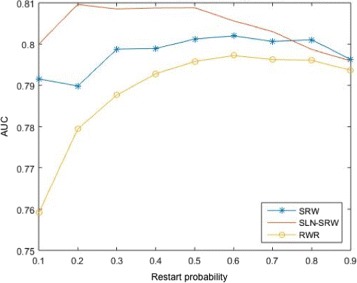
he AUC score for each given restart probability for three methods. The AUC score for each given restart probability for three methods. The red, blue and yellow lines are represent SLN-SRW, SRW and RWR method respectively

**Fig. 5:**
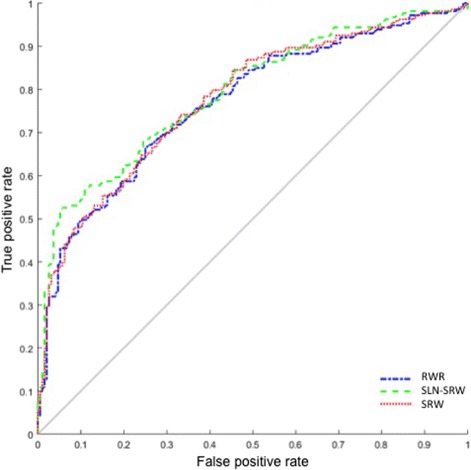
ROC curves for the experimental results on testing set. ROC curves for the experimental results on testing set. ROC curves for the experimental results calculated with SLN-SRW (green), SRW (red) and RWR (blue)

Varying the restart probability *α* from 0.1 to 0.9, the AUC (area under receiver operating characteristic curve) scores [64] of all the three methods are shown in Fig. 4. If *α*=0.2, SLN-SRW method reaches the highest AUC score 0.81, whereas SRW and RWR have the highest AUC scores if *α*=0.6, indicating that SLN-SRW can find the disease genes which are far from the disease node. Based on the edge weights learned using the training data, we predicted the disease-gene relationships in the testing set. We compared the performance of all the three methods using the receiver operating characteristic (ROC) curve. In our test, the AUC score of SLN-SRW (0.79) is the highest (see Fig. 5). Especially, the true positive rate of SLN-SRW is significantly higher than RWR and SRW while its false positive rate keeps low. This is important for disease gene predict, since researchers usually select candidate disease genes with a stringent threshold, which corresponds to a low false positive rate.

**Fig. 6:**
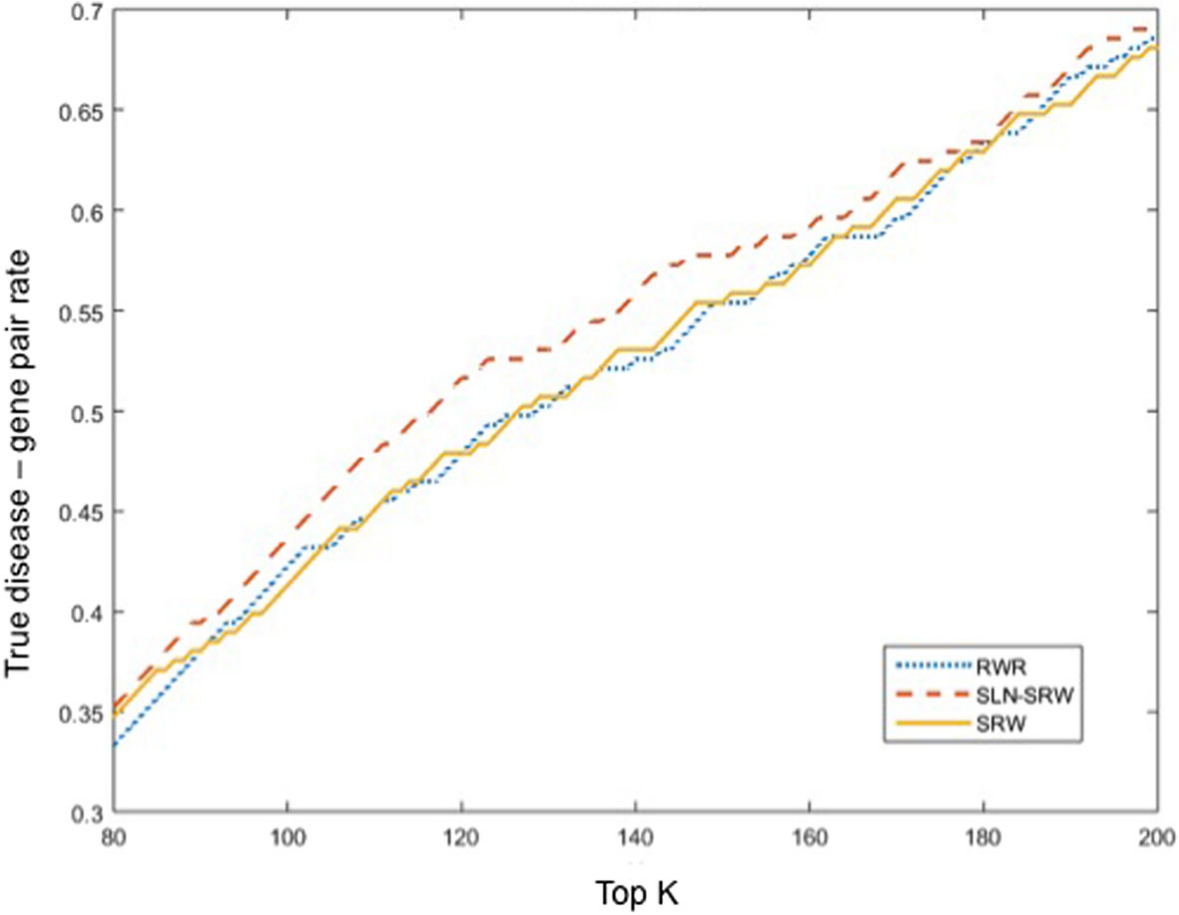
True disease-gene pair rates. True disease-gene pair rates at different top *k* levels

Finally, we ranked the predicted disease genes to check whether the true disease-related genes have higher ranks than the other genes. Figure 6 shows that the prediction result of SLN-SRW contains more known disease-related genes than SRW and RWR at a majority of the top *k* levels, indicating that the edge weighing process in SLN-SRW has contributed significantly to the high recall of the results.

### Performance evaluation on synthetic data set

**Fig. 7:**
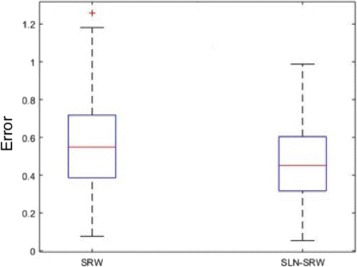
The boxplot of the error score. The boxplot of the error score for SLN-SRW and SRW

To compare SLN-SRW with SRW, we ran both methods on synthetic data, following the method described in below [52]. For SRW and SLN-SRW, we estimated the edge-type parameter based on the synthetic network and the training set described in the “*Data preparation*” subsection, resulting in *w* ^∗^. We measure the performance of SRW and SLN-SRW by comparing the true edge-type parameter *w* ^′^ with *w* ^∗^, using $error = \sum _{i}|w'_{i} - w^{*}_{i}|$. After repeating the experiment 100 times, we find that the error of SLN-SRW is statistically significantly lower than that of SRW (t-test *p*−*value*<0.05) indicating that SLN-SRW performs better than SRW (see Fig. 7). The error of SLN-SRW is also lower in the first and third quartile.

## Conclusions

Identifying the relationships between diseases and genes is vital for disease diagnosis and drug design. Recently, researchers have started to employ integrated biomedical networks to extend the scope and ability for disease gene prediction. In this article, we proposed a novel network-based method named SLN-SRW to define the weight of edges in an integrated network and then use it to predict the gene-disease relationships. SLN-SRW has the following advantages: 1) it can estimate edge weight by differentiating different edge-types; 2) it adopts a Laplacian normalization based method to avoid the bias caused by the super hub nodes in an integrated network; 3) three widely used biomedical ontologies are used to construct a new heterogeneous integrated network. To demonstrate the advantages of SLN-SRW, we compare it with two existing methods SRW and RWR. The experiment on a real data set shows that SLN-SRW performs best among all the three methods. Furthermore, the experiment on a synthetic data set indicates that the edge weights predicted by SLN-SRW are more precise than SRW. Comparing with the existing methods, SLN-SRW has the unique function to identify disease genes, which are not close to any disease node in the disease-gene networks. This could benefit clinicians on discovering new disease-associated genes that have not been identified by the existing methods. Besides, SLN-SRW provides a novel approach to automatically assign weights to the heterogeneous edge types in the disease-gene networks, whereas the existing methods can only define the edge weights manually.

In the future, SLN-SRW will be applied to networks with different edge densities and qualities to test its robustness. Furthermore, we will apply SLN-SRW on more recent datasets and examine the results using both biological experiments and literature.

## Additional files

Additional file 1 Process of mapping different types of IDs. Additional file 1 is a figure to illustrate how different types of IDs are unified. (PDF 54.5 kb)

Additional file 2 Initial weight for difference evidence code. Additional file 2 is a table that lists the weight values for different evidence code. (PDF 42 kb)

Additional file 3 Relation between parameter *b* and loss value. Additional file 3 is a figure showing the relation between parameter *b* and loss value. (PNG 155 kb)

Additional file 4 Diseases selected as the evaluation set. Additional file 4 is a table of diseases selected as the evaluation set. (PDF 708 kb)

## References

[1] Wang X, Gulbahce N, Yu H. (2011). Network-based methods for human disease gene prediction. Brief Funct Genomics.

[2] Ala U, Piro RM, Grassi E, Damasco C, Silengo L, Oti M, Provero P, Di Cunto F (2008). Prediction of human disease genes by human-mouse conserved coexpression analysis. PLoS Comput Biol.

[3] Kann MG (2010). Advances in translational bioinformatics: computational approaches for the hunting of disease genes. Brief Bioinformatics.

[4] Jiang Q, Wang J, Wu X, Ma R, Zhang T, Jin S, Han Z, Tan R, Peng J, Liu G (2015). LncRNA2Target: a database for differentially expressed genes after lncRNA knockdown or overexpression. Nucleic Acids Res.

[5] Navlakha S, Kingsford C (2010). The power of protein interaction networks for associating genes with diseases. Bioinformatics.

[6] Jiang q, Wang G, Zhang T, Wang Y (2013). Predicting human microRNA-disease associations based on support vector machine. Int J Data Mining Bioinformatics.

[7] Browne F, Wang H, Zheng H (2015). A computational framework for the prioritization of disease-gene candidates. BMC Genomics.

[8] Chen B, Li M, Wang J, Shang X, Wu FX (2015). A fast and high performance multiple data integration algorithm for identifying human disease genes. BMC Med Genomics.

[9] Chen B, Shang X, Li M, Wang J, Wu FX (2016). Identifying individual-cancer-related genes by re-balancing the training samples. IEEE Transactions on Nanobioscience.

[10] Jiang q, Hao Y, Wang G, Juan L, Zhang T, Teng M, Liu Y, Wang Y (2010). Prioritization of disease microRNAs through a human phenome-microRNAome network. BMC Syst Biol.

[11] Bush WS, Dudek SM, Ritchie MD (2009). Biofilter: a knowledge-integration system for the multi-locus analysis of genome-wide association studies. Pacific Symposium on Biocomputing. Pacific Symposium on Biocomputing.

[12] Yu S, Van Vooren S, Tranchevent LC, De Moor B, Moreau Y (2008). Comparison of vocabularies, representations and ranking algorithms for gene prioritization by text mining. Bioinformatics.

[13] Aerts S, Lambrechts D, Maity S, Van Loo P, Coessens B, De Smet F, Tranchevent LC, De Moor B, Marynen P, Hassan B (2006). Gene prioritization through genomic data fusion. Nat Biotechnol.

[14] Hu Y, Zhou W, Ren J, Dong L, Wang Y, Jin S, Cheng L. Annotating the function of the human genome with gene ontology and disease ontology. BioMed Res Int. 2016;4130861. 10.1155/2016/4130861 PMC5011202 27635398

[15] Zhang T, Hu Y, Wu X, Ma R, Jiang Q, Wang Y (2016). Identifying liver cancer-related enhancer SNPs by integrating GWAS and histone modification ChIP-seq data. BioMed Res Int.

[16] Peng J, Uygun S, Kim T, Wang Y, Rhee SY, Chen J (2015). Measuring semantic similarities by combining gene ontology annotations and gene co-function networks. BMC Bioinformatics.

[17] Cheng L, Li J, Hu Y, Jiang Y, Liu Y, Chu Y, Wang Z, Wang Y (2015). Using semantic association to extend and infer literature-oriented relativity between terms. IEEE/ACM Trans Comput Biol Bioinformatics.

[18] Cheng L, Jiang Y, Wang Z, Shi H, Sun J, Yang H, Zhang S, Hu Y, Zhou M (2016). DisSim: an online system for exploring significant similar diseases and exhibiting potential therapeutic drugs. Sci Rep.

[19] Peng J, Wang Y, Chen J (2014). Towards integrative gene functional similarity measurement. BMC Bioinformatics.

[20] Peng J, Li H, Jiang Q, Wang Y, Chen J (2014). An integrative approach for measuring semantic similarities using gene ontology. BMC Syst Biol.

[21] Peng J, Li H, Liu Y, Juan L, Jiang q, Wang Y, Chen J (2016). InteGO2: a web tool for measuring and visualizing gene semantic similarities using gene ontology. BMC Genomics.

[22] Schlicker A, Lengauer T, Albrecht M (2010). Improving disease gene prioritization using the semantic similarity of gene ontology terms. Bioinformatics.

[23] Peng J, Wang T, Hu J, Wang YW, Chen J (2016). Constructing Networks of Organelle Functional Modules in Arabidopsis. Curr Genomics.

[24] Cheng L, Shi H, Wang Z, Hu Y, Yang H, Zhou C, Sun J, Zhou M (2016). IntNetLncSim: an integrative network analysis method to infer human lncRNA functional similarity. Oncotarget.

[25] Hu Y, Zhang Y, Ren J, Wang Y, Wang Z, Zhang J. Statistical approaches for the construction and interpretation of human protein-protein interaction network. BioMed Res Int. 2016;5313050. 10.1155/2016/5313050 PMC5015007 27648447

[26] Song S, Hao J, Liu Y, Sun J (2015). Improved EGT-Based Robustness Analysis of Negotiation Strategies in Multiagent Systems via Model Checking. IEEE Trans Human-Mach Syst.

[27] Moreau Y, Tranchevent LC (2012). Computational tools for prioritizing candidate genes: boosting disease gene discovery. Nat Rev Genet.

[28] Barabási AL, Gulbahce N, Loscalzo J (2011). Network medicine: a network-based approach to human disease. Nat Rev Genet.

[29] Schadt EE (2009). Molecular networks as sensors and drivers of common human diseases. Nature.

[30] Oti M, Snel B, Huynen MA, Brunner HG (2006). Predicting disease genes using protein–protein interactions. J Med Genet.

[31] Krauthammer M, Kaufmann CA, Gilliam TC, Rzhetsky A (2004). Molecular triangulation: bridging linkage and molecular-network information for identifying candidate genes in Alzheimer’s disease. Proc Natl Acad Sci U S A.

[32] Köhler S, Bauer S, Horn D, Robinson PN (2008). Walking the interactome for prioritization of candidate disease genes. Am J Hum Genet.

[33] Li Y, Patra JC (2010). Genome-wide inferring gene–phenotype relationship by walking on the heterogeneous network. Bioinformatics.

[34] Vanunu O, Magger O, Ruppin E, Shlomi T, Sharan R (2010). Associating genes and protein complexes with disease via network propagation. PLoS Comput Biol.

[35] Van Dongen S (2008). Graph clustering via a discrete uncoupling process. SIAM J Matrix Anal Appl.

[36] Navlakha S, White J, Nagarajan N, Pop M, Kingsford C. Finding biologically accurate clusterings in hierarchical tree decompositions using the variation of information. In: Research in Computational Molecular Biology. Springer: 2009. p. 400–17. 10.1089/cmb.2009.0173 20377460

[37] Goel R, Harsha H, Pandey A, Prasad TK (2012). Human protein reference database and human proteinpedia as resources for phosphoproteome analysis. Mol bioSystems.

[38] Brown KR, Jurisica I (2005). Online predicted human interaction database. Bioinformatics.

[39] Amberger JS, Bocchini CA, Schiettecatte F, Scott AF, Hamosh A (2015). OMIM. org: Online Mendelian Inheritance in Man (OMIM®;), an online catalog of human genes and genetic disorders. Nucleic Acids Res.

[40] Wang B, Mezlini AM, Demir F, Fiume M, Tu Z, Brudno M, Haibe-Kains B, Goldenberg A (2014). Similarity network fusion for aggregating data types on a genomic scale. Nat Methods.

[41] Wang J, Chen G, Li M, Pan Y (2011). Integration of breast cancer gene signatures based on graph centrality. BMC Syst Biol.

[42] Liekens AM, De Knijf J, Daelemans W, Goethals B, De Rijk P, Del-Favero J (2011). BioGraph: unsupervised biomedical knowledge discovery via automated hypothesis generation. Genome Biol.

[43] Ganegoda GU, Wang J, Wu FX, Li M (2014). Prediction of disease genes using tissue-specified gene-gene network. BMC Syst Biol.

[44] Eronen L, Toivonen H (2012). Biomine: predicting links between biological entities using network models of heterogeneous databases. BMC Bioinformatics.

[45] Groza T, Köhler S, Moldenhauer D, Vasilevsky N, Baynam G, Zemojtel T, Schriml LM, Kibbe WA, Schofield PN, Beck T (2015). The human phenotype ontology: semantic unification of common and rare disease. Am J Hum Genet.

[46] Kibbe WA, Arze C, Felix V, Mitraka E, Bolton E, Fu G, Mungall CJ, Binder JX, Malone J, Vasant D (2015). Disease Ontology 2015 update: an expanded and updated database of human diseases for linking biomedical knowledge through disease data. Nucleic Acids Res.

[47] Consortium GO (2015). Gene ontology consortium: going forward. Nucleic Acids Res.

[48] Peng J, Wang T, Wang J, Wang Y, Chen J (2016). Extending gene ontology with gene association networks. Bioinformatics.

[49] Szklarczyk D, Franceschini A, Wyder S, Forslund K, Heller D, Huerta-Cepas J, Simonovic M, Roth A, Santos A, Tsafou KP (2015). STRING v10: protein–protein interaction networks, integrated over the tree of life. Nucleic Acids Res.

[50] Landrum MJ, Lee JM, Riley GR, Jang W, Rubinstein WS, Church DM, Maglott DR (2014). ClinVar: public archive of relationships among sequence variation and human phenotype. Nucleic Acids Res.

[51] Cheng L, Wang G, Li J, Zhang T, Xu P, Wang Y (2013). SIDD: a semantically integrated database towards a global view of human disease. PloS ONE.

[52] Backstrom L, Leskovec J (2011). Supervised random walks: predicting and recommending links in social networks. Proceedings of the fourth ACM international conference on Web search and data mining.

[53] Johnson R, Zhang T (2007). On the Effectiveness of Laplacian Normalization for Graph Semi-supervised Learning. J Mach Learn Res.

[54] Tong H, Faloutsos C, Pan JY (2008). Random walk with restart: fast solutions and applications. Knowl Inf Syst.

[55] Mattingly C, Rosenstein M, Colby G, Forrest J, Boyer J (2006). The Comparative Toxicogenomics Database (CTD): a resource for comparative toxicological studies. J Exp Zool Part A Comparative Exp Biol.

[56] Hamosh A, Scott AF, Amberger JS, Bocchini CA, McKusick VA (2005). Online Mendelian Inheritance in Man (OMIM), a knowledgebase of human genes and genetic disorders. Nucleic Acids Res.

[57] Povey S, Lovering R, Bruford E, Wright M, Lush M, Wain H (2001). The HUGO gene nomenclature committee (HGNC). Hum Genet.

[58] Lipscomb CE (2000). Medical subject headings (MeSH). Bull Med Libr Assoc.

[59] Bodenreider O (2004). The unified medical language system (UMLS): integrating biomedical terminology. Nucleic Acids Res.

[60] Schriml LM, Arze C, Nadendla S, Chang YWW, Mazaitis M, Felix V, Feng G, Kibbe WA (2012). Disease Ontology: a backbone for disease semantic integration. Nucleic Acids Res.

[61] Köhler S, Doelken SC, Mungall CJ, Bauer S, Firth HV, Bailleul-Forestier I, Black GC, Brown DL, Brudno M, Campbell J (2014). The Human Phenotype Ontology project: linking molecular biology and disease through phenotype data. Nucleic Acids Res.

[62] Ashburner M, Ball CA, Blake JA, Botstein D, Butler H, Cherry JM, Davis AP, Dolinski K, Dwight SS, Eppig JT (2000). Gene Ontology: tool for the unification of biology. Nat Genet.

[63] Kumar R, Raghavan P, Rajagopalan S, Sivakumar D, Tomkins A, Upfal E (2000). Stochastic models for the web graph. Foundations of Computer Science, 2000. Proceedings. 41st Annual Symposium on.

[64] Hanley JA, McNeil BJ (1982). The meaning and use of the area under a receiver operating characteristic (ROC) curve. Radiology.

