# Supplementary figures and images for: Predicting disease-related genes using integrated biomedical networks
Source: BMC Genomics. 2017 Jan 25;18(Suppl 1):1043. doi: 10.1186/s12864-016-3263-4 (PMC5310285; doi:10.1186/s12864-016-3263-4)

Additional File 1: Process of mapping different types of IDs

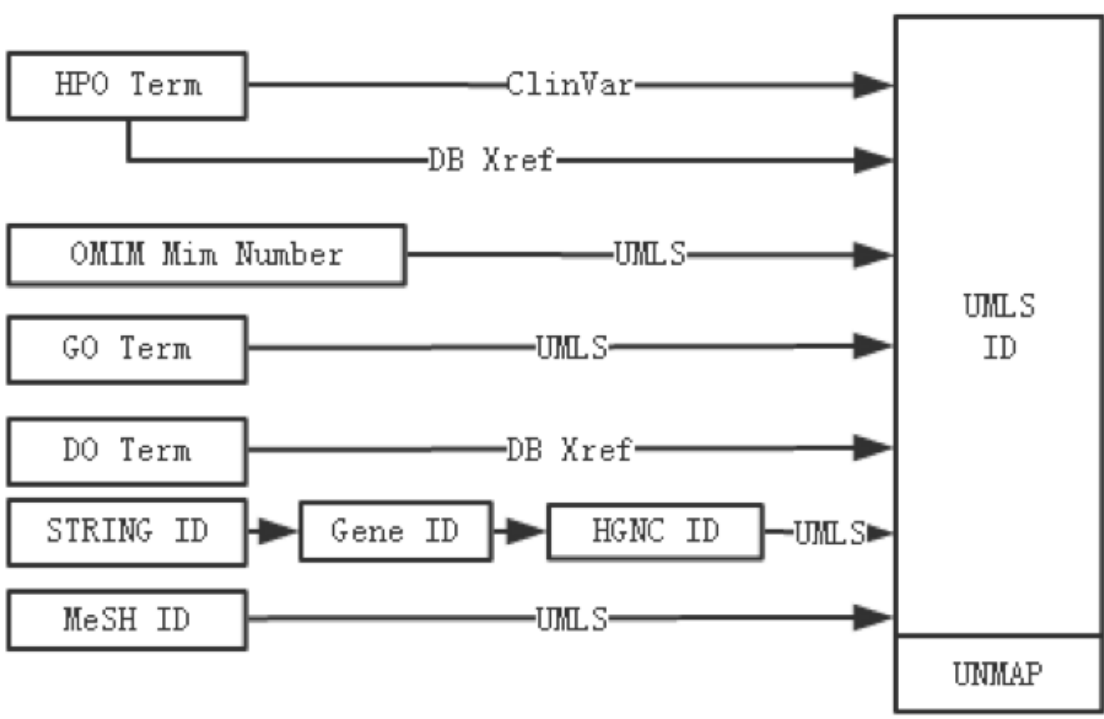

Supplement: Additional file 1 — Process of mapping different types of IDs. Additional file 1 is a figure to illustrate how different types of IDs are unified. (PDF 54.5 kb) [file 12864_2016_3263_MOESM1_ESM.pdf]

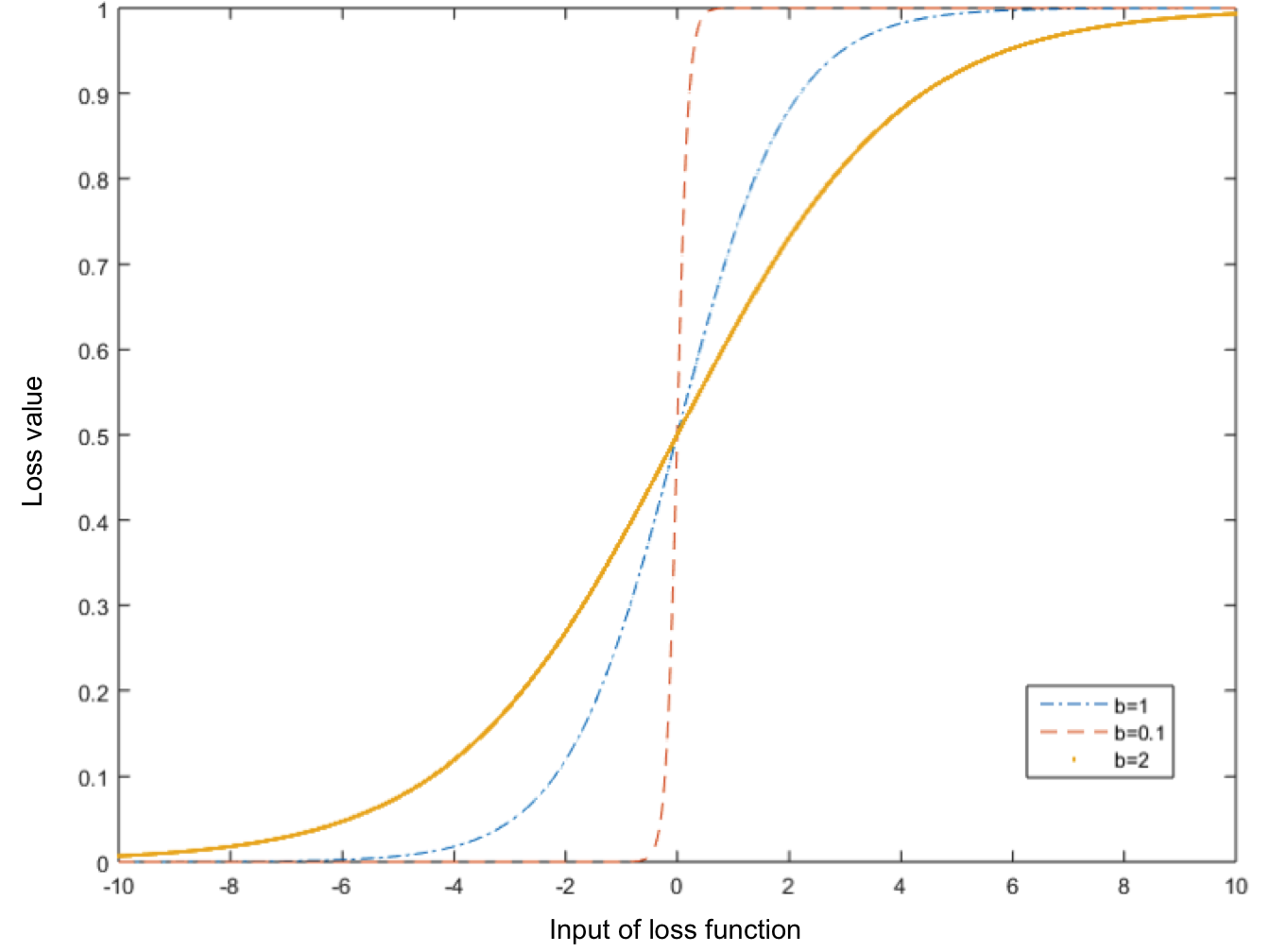

Supplement: Additional file 3 — Relation between parameter b and loss value. Additional file 3 is a figure showing the relation between parameter b and loss value. (PNG 155 kb) [file 12864_2016_3263_MOESM3_ESM.png]
