# Supplementary material for: Predicting disease-related genes using integrated biomedical networks
Source: BMC Genomics. 2017 Jan 25;18(Suppl 1):1043. doi: 10.1186/s12864-016-3263-4 (PMC5310285; doi:10.1186/s12864-016-3263-4)
Supplement: Additional file 2 — Initial weight for difference evidence code. Additional file 2 is a table that lists the weight values for different evidence code. (PDF 42 kb) [file 12864_2016_3263_MOESM2_ESM.pdf]

Additional file 2: Initial weight for different evidence codes

Data source	Evidence code	Abr. Of Evidence code	Number	Initial weight
SIDD,CTD	do_sidd_mesh_gene_MFR	DMR	17072	0.8
	do_sidd_mesh_gene_MFS	DMS	1097	0.8
	do_sidd_mesh_gene_MFI	DMI	3756	0.6
SIDD,OMIM	do_sidd_omim_gene_MFR	DOR	1872	0.8
	do_sidd_omim_gene_MFS	DOS	257	0.8
	do_sidd_omim_gene_MFI	DOI	2472	0.6
OMIM	do_xref_omim_gene	DXM	666	1.0
ClinVar	disease_clinvar_gene	DVG	21167	0.8
CTD	disease_ctd_gene_curated	DCG	3559	0.8
STRING	string_gene	SGG	102962	Weight in STRING
GOA	go_annotation_gene_EXP	GEXP	221	1.0
	go_annotation_gene_IDA	GIDA	55469	1.0
	go_annotation_gene_IPI	GIPI	12604	1.0
	go_annotation_gene_IMP	GIMP	13781	1.0
	go_annotation_gene_IGI	GIGI	679	1.0
	go_annotation_gene_IEP	GIEP	929	1.0
	go_annotation_gene_IEA	GIEA	79772	0.4
	go_annotation_gene_ND	GND	1892	0.4
	go_annotation_gene_IC	GIC	1235	0.6
	go_annotation_gene_NAS	GNAS	6935	0.6
	go_annotation_gene_TAS	GTAS	47951	0.8
	go_annotation_gene_IKR	GIKR	29	0.6
	go_annotation_gene_IRD	GIRD	2	0.6
	go_annotation_gene_IBA	GIBA	13559	0.6
	go_annotation_gene_ISS	GISS	17573	0.6
HPO	hp_annotation_gene_IEA	HIEA	2637	0.4
OMIM	hp_annotation_gene_ICE	HICE	1	0.8
	hp_annotation_gene_PCS	HPCS	105	0.8
	hp_annotation_gene_TAS	HTAS	1500	0.8
GO	go_is_a	GOA	72459	1.0
	go_part_of	GOP	8527	0.8
	go_regulates	GOR	3208	0.6
DO	do_is_a	DOA	6919	1.0
HP	hp_is_a	HPA	14762	1.0
