# Supplementary material for: Predicting disease-related genes using integrated biomedical networks
Source: BMC Genomics. 2017 Jan 25;18(Suppl 1):1043. doi: 10.1186/s12864-016-3263-4 (PMC5310285; doi:10.1186/s12864-016-3263-4)
Supplement: Additional file 4 — Diseases selected as the evaluation set. Additional file 4 is a table of diseases selected as the evaluation set. (PDF 708 kb) [file 12864_2016_3263_MOESM4_ESM.pdf]

Additional file 4: Diseases selected as the evaluation set

Disease name	ID	Ontology ID	Number of genes associated with disease
Aneima	C0002871	DOID:2355	23
Hemolytic anemia	C0002878	DOID:583,HP:0001878	14
Cardiomyopathies	C0878544	HP:0001638	46
Charcot-Marie-Tooth Disease	C0007959	DOID:10595	21
Diabetes	C0011849	DOID:9351	20
Dystonia	C0013421	HP:0001332	13
Leukemia	C0023418	DOID:1240, HP:0001909	32
Lymphoma	C0024299	HP:0002665	17
Muscular Dystrophy	C0026850	DOID:9884, HP:0003560	9
Myopathy	C0026848	DOID:423, HP:0003198	27
Parkinson Disease	C0030567	DOID:14330	72
Retinitis Pigmentosa	C0035334	HP:0000510	67
Spastic Paraplegia	C0037773	DOID:2476	35
Usher Syndromes	NMAP12546	DOID:0050439	12
Xeroderma Pigmentosum	NMAP12777	DOID:0050427	8
Zellweger Syndrome	C0043459	DOID:905	14
